# A hierarchical prognostic model for risk stratification in patients with early breast cancer according to ^18^F‐fludeoxyglucose uptake and clinicopathological parameters

**DOI:** 10.1002/cam4.1394

**Published:** 2018-02-26

**Authors:** Jongtae Cha, Hyung Seok Park, Dongwoo Kim, Hyun Jeong Kim, Min Jung Kim, Young Up Cho, Mijin Yun

**Affiliations:** ^1^ Department of Nuclear Medicine Severance Hospital Yonsei University College of Medicine Seoul Korea; ^2^ Department of General Surgery Severance Hospital Yonsei University College of Medicine Seoul Korea; ^3^ Department of Radiology Severance Hospital Yonsei University College of Medicine Seoul Korea

**Keywords:** ^18^F‐FDG PET/CT, classification and regression tree modeling, early breast cancer, recurrence‐free survival, risk model

## Abstract

This study was to investigate a hierarchical prognostic model using clinicopathological factors and ^18^F‐fludeoxyglucose (FDG) uptake on positron emission tomography/computed tomography (PET/CT) for recurrence‐free survival (RFS) in patients with early breast cancer who underwent surgery without neoadjuvant chemotherapy. A total of 524 patients with early breast cancer were included. The Cox proportional hazards model was used with clinicopathological variables and maximum standardized uptake value (SUVmax) on PET/CT. After classification and regression tree (CART) modeling, RFS curves were estimated using the Kaplan–Meier method and differences in each risk layer were assessed using the log‐rank test. During a median follow‐up of 46.2 months, 31 (5.9%) patients experienced recurrence. The CART model identified four risk layers: group 1 (SUVmax ≤6.75 and tumor size ≤2.0 cm); group 2 (SUVmax ≤6.75 and Luminal A [LumA] or TN tumor >2.0 cm); group 3 (SUVmax ≤6.75 and Luminal B [LumB] or human epidermal growth factor receptor 2 [HER2]‐enriched] tumor >2.0 cm); group 4 (SUVmax >6.75). Five‐year RFS was as follows: 95.9% (group 1), 98% (group 2), 82.8% (group 3), and 85.4% (group 4). Group 3 or group 4 showed worse prognosis than group 1 or group 2 (group 1 vs. group 3: *P *= 0.040; group 1 vs. group 4: *P* < 0.001; group 2 vs. group 3: *P* = 0.016; group 2 vs. group 4: *P* < 0.001). High SUVmax (>6.75) in primary breast cancer was an independent factor for poor RFS. In patients with low SUVmax, LumB or HER2‐enriched tumor >2 cm was also prognostic for poor RFS, similar to high SUVmax.

## Introduction

Breast cancer is the most common malignancy among women worldwide 1. Due to increased screening, the majority of patients present with early‐stage disease 2. Treatment of choice in early breast cancer (stages 0, 1, and 2) is surgical resection followed by adjuvant chemotherapy according to clinicopathological risk factors 3, 4. The known prognostic factors are axillary nodal status, tumor size, tumor pathology, tumor grade, peritumoral lymphatic vessel and vascular invasion, hormonal receptors, proliferation markers, ethnicity, and age, among others 5. In addition, five intrinsic subtypes based on gene expression profiles have shown distinct tumor phenotypes in predicting patient outcomes 6, 7. Genomic risk stratification has been also useful in selecting patients who would benefit from adjuvant chemotherapy 8. Due to limitations preventing the routine use of gene expression profiling, immunohistochemistry (IHC) staining for hormonal receptors and human epidermal growth factor receptor 2 (HER2) has been preferred as a therapy‐oriented surrogate of intrinsic subtype 9, 10.

^18^F‐fludeoxyglucose (FDG) positron emission tomography/computed tomography (PET/CT) has advantages over conventional imaging modalities in that it offers noninvasive, semiquantitative information about metabolically active tumor burden, tumor biology, and patient prognosis 11, 12, 13. Unlike pathological and genomic prognostic factors, clinically relevant information can be provided in preoperative settings with potential therapeutic implications. Although maximum standardized uptake value (SUVmax) in primary breast cancer has been found to be an independent prognostic factor for survival, studies have included patient populations with different risks for recurrence by including both early and locally advanced cancers, with or without neoadjuvant chemotherapy. Nevertheless, a risk stratification model using metabolic variables on PET/CT combined with other known prognostic factors has not been proposed.

The purpose of this study was to investigate the prognostic value of ^18^F‐FDG uptake in the primary tumor for recurrence‐free survival (RFS) in patients with early breast cancer who underwent surgery without neoadjuvant chemotherapy. In addition to conventional statistical modeling of prognosis, a hierarchical prognostic system was applied to identify further synergistic/antagonistic interactions between clinicopathological prognostic factors and ^18^F‐FDG uptake.

## Materials and Methods

### Patients

**Figure 1:**
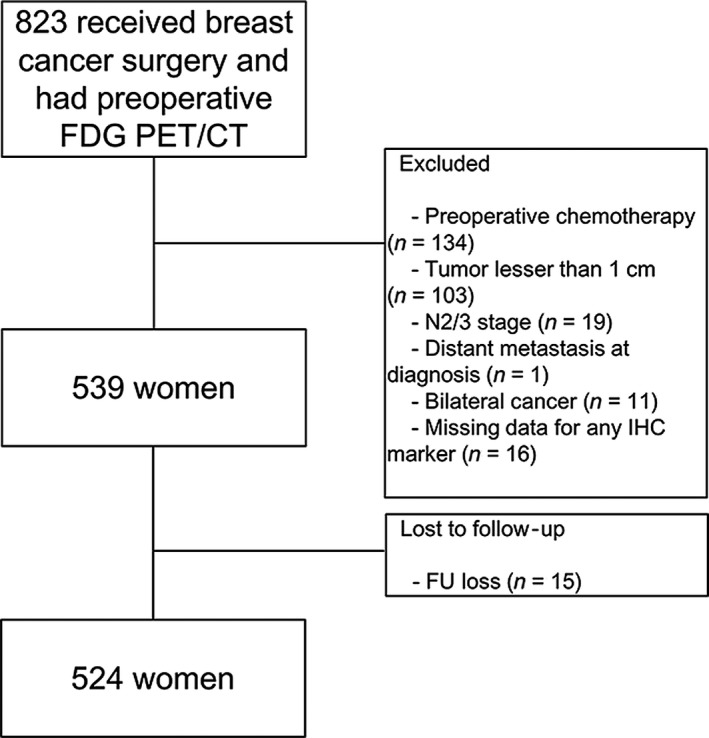
Flow diagram of patients included in the current study. FDG PET/CT indicates ^18^F‐fludeoxyglucose positron emission tomography/computed tomography; IHC immunohistochemistry.

Data from patients with breast cancer who underwent ^18^F‐FDG PET/CT at initial staging between January 2008 and December 2013 were retrieved from the institutional medical database. Patients were included if they had pathologically proven breast cancer and IHC results for estrogen receptor (ER), progesterone receptor (PR), HER2, and Ki‐67 status. To be included, patients also had to have undergone mastectomy with sentinel lymph node dissection and/or axillary lymph node dissection without neoadjuvant treatment. Among these, patients with locally advanced breast cancer (N2/3 classified according to 7th edition of American Joint Committee on Cancer [AJCC])^14^ were excluded. Tumors <1 cm in size were excluded to minimize a partial‐volume averaging effect affecting semiquantitative measurement of ^18^F‐FDG uptake. Patients who had bilateral breast cancers were excluded. Patients visited the hospital every 3 months for the first year of follow‐up. Routine examinations, including breast sonography, mammography, and whole body bone scan, were performed every 6 months for the first year and annually thereafter. Whenever abnormal findings were noted, further diagnostic studies were performed for confirmation. Patients without regular follow‐up data were excluded. Finally, 524 patients were included in the analysis (Fig. 1). This study was approved by the Institutional Review Board of our Hospital. Given the retrospective nature of this study and the use of anonymized data, the requirement for informed consent was waived.

### PET/CT imaging

PET/CT was performed using a scanner (Discovery STE; GE Healthcare, Milwaukee, WI or Biograph TruePoint 40; Siemens Medical Systems, Knoxville, TN) with 16‐ or 40‐slice CT, respectively. Patients fasted for at least 6 h before imaging, and glucose levels in the peripheral blood were ≤140 mg/dL before the injection of ^18^F‐FDG. An ^18^F‐FDG dose of approximately 5.5 MBq/kg was administered intravenously 1 h before image acquisition. After the initial low‐dose CT study (Discovery STE, 30 mA, 130 kVp or Biograph TruePoint 40, 36 mA, 120 kVp), a standard PET protocol was used to scan from the neck to the proximal thighs with an acquisition time of 2.5 min per bed position in the three‐dimensional mode. PET images were reconstructed using ordered subset expectation maximization with CT images for attenuation correction.

### Measurement of PET parameters

Two experienced nuclear medicine physicians reviewed all PET/CT images; discrepancies were resolved by consensus. Metabolic parameters were measured using Volume Viewer software (GE Healthcare, USA). For semiquantitative analysis, a volume of interest was drawn for each lesion to measure SUVmax. SUVmax was calculated using the following formula: (decay‐corrected activity [kBq]/tissue volume [mL])/(injected ^18^F‐FDG activity [kBq]/body mass [g]).

### Histologic evaluation and categorization of molecular subtypes

All patients underwent surgical resection for breast cancer with sentinel lymph node biopsy and/or axillary lymph node dissection. Histologic type, tumor size, tumor grade, and lymphovascular invasion (LVI) status were determined from the surgically excised specimens. Tumor, node, and metastasis (TNM) staging was performed according to the 7th edition of the AJCC 14. The histologic grade of each tumor was determined using the modified Bloom–Richardson classification 15. Formalin‐fixed, paraffin‐embedded tissue blocks were used for IHC; the expression status of ER, PR, HER2, and Ki‐67 was determined by IHC staining of the surgical specimen. Primary antibodies against ER (Clone SP1; Neomarkers for Lab Vision, Fremont, USA), PR (Clone PgR 636; DAKO, Glostrup, Denmark), HER2 (Clone Polyclonal; DAKO, Glostrup, Denmark), Ki67 (Clone MIB‐1; DAKO, Glostrup, Denmark) were used. ER positivity (+) and PR (+) were defined as the presence of ≥1% positively stained nuclei in 10 high‐power fields. HER2 staining was analyzed according to the American Society of Clinical Oncology/College of American Pathologists guidelines^16^. The intensity of HER2 staining was scored as 0, 1+, 2+, or 3+. Tumors with a 3+ score were considered to be HER2 (+), whereas those with a 0 or 1+ score were considered to be HER2 negative (−). Tumors with a 2+ score were subjected to fluorescence in situ hybridization analysis to determine HER2 status. For Ki‐67, nuclear staining ≥14% was considered to be high‐level expression. According to different combinations of ER, PR, HER2, and Ki‐67, the patients were categorized into four different molecular subtypes as follows: Luminal A (LumA): ER (+) and/or PR (+), HER2 (−), and Ki‐67 low (<14%); Luminal B (LumB): ER (+) and/or PR (+), HER2 (−), and Ki‐67 high (≥14%) or ER (+) and/or PR (+), HER2 (+), and any Ki‐67 index; HER2‐enriched: ER (−), PR (−), but HER2 (+); and triple negative (TN): ER (−), PR (−), and HER2 (−).

### Statistical analysis

RFS was defined as the time from the operation date to the date of cancer recurrence. The prognostic values of clinicopathological factors, including age, tumor size, nodal status, TNM stage, histologic type, histologic grade, LVI, molecular subtypes, proliferation index, treatment modalities, and SUVmax, were assessed using a Cox proportional hazards model for RFS. Hazard ratio (HR) with Wald 95% confidence intervals (CI) was provided for the model. All continuous variables were grouped into two categories according to the optimal cutoff values determined using receiver operating characteristic (ROC) curve analysis. Multicollinearity among independent variables was evaluated using Spearman rank correlation coefficient before multivariate analysis.

Next, to identify further synergistic/antagonistic interactions between prognostic factors, a classification and regression tree (CART) was generated for RFS. Left‐truncated and right‐censored (LTRC) CART is a prognostic system with a hierarchical structure based on recursive partitioning that builds a decision tree to find subgroups at higher risk for recurrence. Factors that were statistically significant in univariate analyses were included in the CART analysis. Each node on the decision trees included subgroups of a population with common characteristics influencing RFS. HRs of each group elicited by CART model were assessed using Cox proportional hazard model. Finally, cumulative RFS curves of each risk layer were estimated using the Kaplan–Meier method, and the statistical differences among the risk layers were compared using the log‐rank test with pairwise comparison to account for multiple comparison correction.

All statistical analyses were performed using SPSS version 23 (IBM Corporation, Armonk, NY, USA) for Windows (Microsoft Corporation, Redmond, WA, USA) and R 3.33.0 software (The R Foundation for Statistical Computing, Vienna, Austria) with necessary analytical packages such as LTRC trees, rpart, and survival; *P* < 0.05 was considered to be statistically significant.

## Results

### Patient characteristics

**Figure 2:**
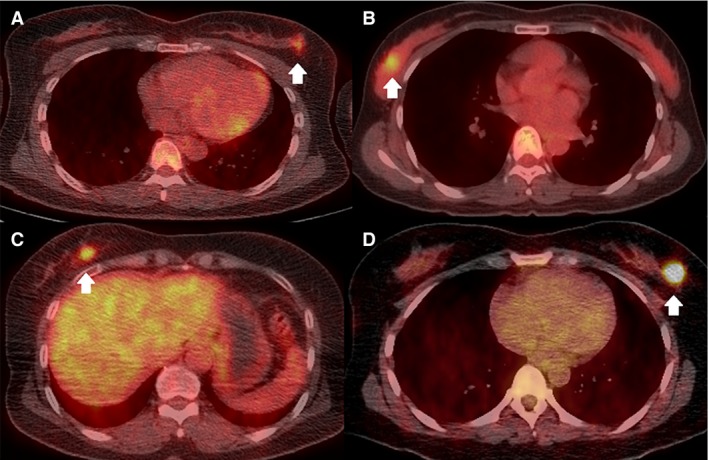
Representative ^18^F‐fludeoxyglucose positron emission tomography/computed tomography images of patients in each molecular subtype. (A) Luminal A subtype (SUVmax = 3.30, tumor size = 1.8 cm); (B) Luminal B subtype (SUVmax = 5.86, tumor size = 2.0 cm); (C) human epidermal growth factor receptor 2‐enriched subtype (SUVmax = 6.80, tumor size = 1.9 cm); (D) triple negative subtype (SUVmax = 9.50, tumor size = 1.8 cm). SUVmax indicates maximum standardized uptake value.

The clinicopathological characteristics of the 524 female patients (median age 50 years [range, 27–82 years]) are summarized in Table 1. There were 270 of 524 (51.5%) patients with TNM stage I and 254 of 524 (48.5%) with stage II cancer. Of the 125 of 524 (23.9%) patients with lymph node metastasis (N1), 23 of 524 (4.4%) had known N1 metastasis according to ultrasonography‐guided biopsy before surgery, and 102 of 524 (19.5%) exhibited lymph node metastasis only by sentinel lymph node sampling. There were 225 of 524 (42.9%) patients with LumA, 155 of 524 (29.6%) with LumB, 42 of 524 (8.0%) with HER2‐enriched, and 102 of 524 (19.5%) with TN subtypes. The mean SUVmax of the primary tumors was 5.51 ± 4.10. Representative PET/CT images of patients in each molecular subtype are shown in Figure 2.

**Table 1:** Patient characteristics

Variable	
Age, median year [range]	50 (27–82)
SUVmax (g/mL)	5.51 ± 4.10
Tumor size (cm)	1.92 ± 0.79
Nodal status (n)	
Negative	399 (76.1%)
Positive	
Sentinel LN positive	102 (19.5%)
Axillary LN positive	23 (4.4%)
TNM stage (n)	
I/II	270 (51.5%)/254 (48.5%)
Histologic type (n)	
IDC/ILC/Other^a^	457 (87.2%)/16 (3.1%)/51 (9.7%)
Histologic grade (n)	
1/2/3	110 (21.0%)/240 (45.8%)/174 (33.2%)
Lymphovascular invasion (n)	
Negative/Positive	465 (88.7%)/59 (11.3%)
Ki67 (%)	21.78 ± 21.70
Molecular subtype (n)	
LumA/LumB/HER2‐enriched/TN	225 (42.9%)/155 (29.6%)/42 (8.0%)/102 (19.5%)
Adjuvant chemotherapy (n)	
Done/Not done	430 (82.1%)/94 (17.9%)

LumA, Luminal A; LumB, Luminal B; HER2, human epidermal growth factor receptor 2; TN, triple negative.

Data are reported as mean with standard deviation unless otherwise specified.

a Other: primary breast cancer other than invasive ductal carcinoma (IDC) or invasive lobular carcinoma (ILC).

During a median follow‐up duration of 46.2 months (range, 5.4–95.2 months), 31 of 524 (5.9%) patients experienced recurrence (8 locoregional recurrences, 23 distant metastases). Fourteen of the 270 patients with stage I and 17 of the 254 patients with stage II cancer had recurrence. According to molecular subtype, there was recurrence in 8 of 225 (3.6%) with LumA, 10 of 155 (6.5%) with LumB, 4 of 42 (9.5%) with HER2‐enriched, and 9 of 102 (8.8%) with TN.

### Clinicopathological and imaging prognostic factors for RFS

Optimal cutoff values for SUVmax and size were >6.75 and >2 cm, respectively. In univariate analysis, SUVmax (>6.75), size (>2.0 cm), and TN subtype were significant prognostic factors for RFS. In multivariate analysis, SUVmax was the only independent prognostic factor for RFS (HR 2.6 [CI, 1.1–6.1]; *P* = 0.028) (Table 2).

**Table 2:** Univariate and Multivariate Analysis of Recurrence‐free Survival

Variable	Univariate analysis		Multivariate analysis	
	*P*‐value	Hazard ratio (95% CI)	*P*‐value	Hazard ratio (95% CI)
Age (≤35 vs. >35 years)	0.699	0.75 (0.17–3.16)		
SUVmax (≤6.75 vs. >6.75)	0.001	3.50 (1.69–7.23)	0.028	2.60 (1.10–6.14)
Tumor size (≤2 cm vs. >2 cm)	0.019	2.33 (1.15–4.74)	0.205	1.63 (0.76–3.46)
Nodal status (Negative vs. Positive)	0.750	0.74 (0.30–1.82)		
TNM Stage (I vs. II)	0.544	1.24 (0.61–2.52)		
Histologic type				
IDC		1.00		
ILC	0.97	0.00		
Other^a^	0.52	1.40 (0.49–4.02)		
Histologic grade (1 or 2 vs. 3)	0.327	1.42 (0.70–2.91)		
LVI (Negative vs. Positive)	0.830	1.13 (0.39–3.24)		
Ki67 (≤14% vs. >14%)	0.054	2.09 (0.98–4.45)		
Molecular subtype				
LumA		1.00		1.00
LumB	0.183	1.18 (0.74–4.76)	0.658	1.24 (0.46–3.33)
HER2‐enriched	0.108	2.67 (0.80–8.91)	0.529	1.51 (0.41–5.49)
TN	0.046	2.63 (1.01–6.83)	0.579	1.35 (0.46–3.91)
Adjuvant chemotherapy (Done vs. Not done)	0.483	1.45 (0.50–4.16)		

CI, Confidence interval; IDC, invasive ductal carcinoma; ILC, invasive lobular carcinoma; LVI, lymphovascular invasion; LumA, Luminal A; LumB, Luminal B; HER2, human epidermal growth factor receptor 2; TN, triple negative; vs. , versus.

a Other: primary breast cancer other than IDC or ILC.

### Risk stratification using CART analysis

**Figure 3:**
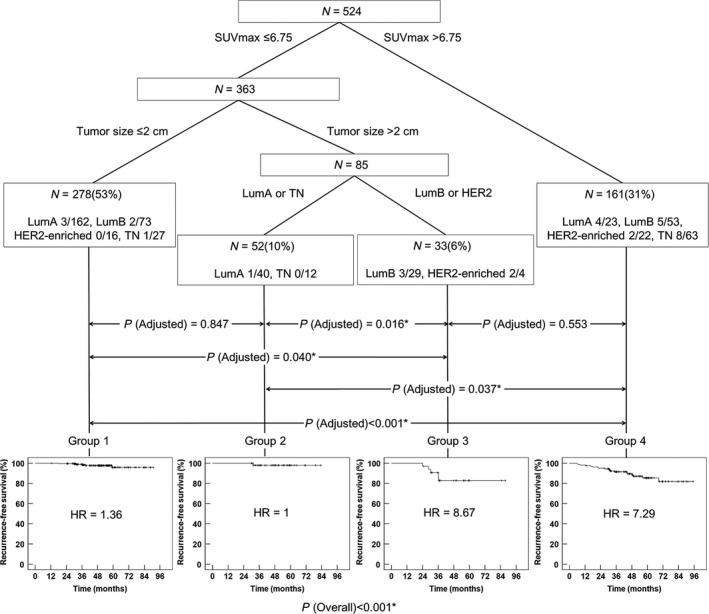
Classification and regression tree analyses to identify synergistic/antagonistic associations between prognostic factors. Square boxes indicate, respectively, intermediate and terminal subsets of patients defined by the sequential splitting process. There are four terminal risk groups. The numbers after molecular subtype are the number of recurrences of the total number of patient in each subtype. Cox proportional hazard model calculated HRs of each group. * = statistically significant difference for pairwise comparison using log‐rank test. HER2 = human epidermal growth factor receptor 2; LumA = Luminal A; LumB = Luminal B; TN = triple negative.

CART modeling was performed using statistically significant variables found in the univariate analysis to identify subgroups at higher risk for recurrence. Accordingly, SUVmax >6.75, tumor size >2.0 cm, and molecular subtypes were included in CART analysis. The resulting CART model revealed four risk layers based on SUVmax, tumor size, and molecular subtype: group 1 (SUVmax ≤6.75 and tumor size ≤2.0 cm); group 2 (SUVmax ≤6.75, tumor size >2.0 cm, and LumA or TN); group 3 (SUVmax ≤6.75, tumor size >2.0 cm, and LumB or HER2‐enriched); group 4 (SUVmax >6.75) (Fig. 3). There were 278 (53%) patients in group 1, 52 (10%) in group 2, 33 (6%) in group 3, and 161 (31%) in group 4. LumA was the most common subtype in group 1, and TN was the most common in group 4.

### Kaplan–Meier survival analysis in four risk layers on CART modeling

According to the risk layers, the 5‐year RFS was 95.9% in group 1, 98% in group 2, 82.8% in group 3, and 85.4% in group 4 (Fig. 3). Group 3 or group 4 demonstrates worse prognosis than group 1 or group 2 (group 1 vs. group 3: *P* = 0.040 after adjustment; group 1 vs. group 4: *P* < 0.001 after adjustment; group 2 vs. group 3: *P* = 0.016 after adjustment; group 2 vs. group 4: *P* < 0.001 after adjustment). No significant difference in RFS was observed between patients in group 1 and those in group 2 (*P* = 0.553 after adjustment). The patients in group 3 showed no remarkable difference in RFS compared with those in group 4 (*P* = 0.847 after adjustment).

## Discussion

Because the majority of patients with breast cancer present at an early stage of disease, we included only a large population of stage I and II breast cancer patients who underwent upfront surgery without neoadjuvant chemotherapy 2, 3. High SUVmax (> 6.75) in the primary tumor was the only independent prognostic factor for RFS in multivariate analysis. Other than SUVmax, tumor size and molecular subtype were significant in univariate analysis. In general, nodal status seems to be the most powerful prognostic factor for RFS in early‐stage breast cancer 17, 18. Regardless, it was not prognostic for RFS in this study. There were 125 of 524 (23.9%) patients with N1 metastasis, in which only a small number (*n *= 23/524 [4.4%]) had N1 metastasis confirmed before surgery. The remaining 102 of 524 (19.5%) patients had N1 disease according to sentinel lymph node sampling. Most N1 metastasis was not clinically detectable, and improved surgical removal of metastatic lymph nodes and effective adjuvant therapy may have contributed to the result.

SUVmax at staging has been prognostic for RFS in patients with operable breast cancer 19, 20, 21, 22, 23, 24, 25. However, no studies have evaluated the interactions between clinicopathological and metabolic prognostic factors for risk stratification. We used CART analysis to evaluate high‐order associations among the significant factors in univariate analysis for further risk stratification. SUVmax >6.75, tumor size >2.0 cm, and molecular subtypes were included in the analysis, resulting in four different risk layers. Among the four risk groups based on CART modeling, patients in groups 3 and 4 showed no significant difference in 5‐year RFS rates (82.8% and 85.4%, respectively). Patients in groups 1 and 2 also showed no difference in 5‐year RFS rates (95.9% and 98%, respectively). The 5‐year RFS rates in group 3 or group 4 were significantly worse than those in group 1 or group 2.

As expected in multivariate analysis, high SUVmax was the highest‐order risk factor for RFS on CART modeling. In group 4 (SUVmax >6.75), neither size nor molecular subtype was significant for further risk classification. This group demonstrated a higher HR (7.29) for RFS. In the remaining patients (groups 1, 2, and 3) with low SUVmax (≤6.75), tumor size was the next classification factor. Patients with a tumor size ≤2 cm represented a separate risk layer with a lower HR (1.36) for RFS (group 1). In patients with tumor size >2 cm, there were two different risk layers according to molecular subtype. In patients with LumA or TN subtypes (group 2), the HR (1.00) was similar to that of group 1, whereas it was 8.67 in patients with LumB or HER2‐enriched subtypes (group 3), similar to that of group 4. Similar to high SUVmax, LumB or HER2‐enriched breast cancers >2 cm appeared to be important in predicting worse RFS in patients with low SUVmax.

TN breast cancer is known to demonstrate the worst survival, whereas LumA has the best survival 26. However, there were interesting risk stratification patterns in patients with TN or LumA subtype in this study. Despite the known poor prognosis of the TN subtype, of the 102 patients with TN, 39 (38%) with low SUVmax showed significantly better RFS than the remaining 63 (62%) patients with high SUVmax. In contrast to LumB or HER2‐enriched, tumor size was not prognostic in TN when SUVmax was low. Given that TN subtype demonstrates heterogeneous histology as well as gene expressions 27, SUVmax appeared to have potential in differentiating patients with different risks for RFS in TN subtype.

In the LumA subtype, there is a high clinical demand for techniques to identify patients who could benefit from toxic adjuvant chemotherapy. Oncotype DX assay (Genomic Health), based on the expression of 21 genes, has been useful in scoring the likelihood of distant metastasis and aiding in treatment decisions 28. In this study, only 23 (10.2%) of the 225 patients with LumA demonstrated high SUVmax and worse RFS. The remaining 202 (89.8%) patients demonstrated low SUV max and better RFS. Similar to the TN subtype, tumor size was not prognostic in LumA when SUVmax was low. Further studies are needed to determine the value of SUVmax to predict RFS and to select patients for adjuvant chemotherapy in LumA breast cancer.

The present study had several limitations, the first of which was its retrospective design. However, we analyzed a large homogenous population of patients with stage I and stage II breast cancer with complete pathological results. Second, we excluded patients whose primary tumor diameter was <1 cm to avoid a partial‐volume averaging effect on SUVmax measurement, which may have resulted in exaggerated prognostic value of SUVmax on small size tumors 29, 30. Even with partial‐volume correction, overcorrection can be problematic in tumors with diameters <1.5 times the full‐width at half‐maximum 31. Regardless, performing ^18^F‐FDG PET/CT in patients with breast cancer tumors <1 cm is less likely.

## Conclusion

In this study, we proposed a risk stratification model using clinicopathological and metabolic prognostic factors in stage I and II breast cancer patients who underwent surgery without neoadjuvant chemotherapy. High SUVmax (>6.75) in the primary tumor was an independent prognostic factor for poor RFS. In patients with low SUVmax, LumB or HER2‐enriched tumor >2 cm was a poor prognostic factor for RFS, similar to high SUVmax.

## Conflict of Interest

This work was supported partially by a National Research Foundation of Korea grant funded by the Korean Government (MSIP) (NRF‐2011‐0030086) and the Basic Science Research Program through the National Research Foundation of Korea funded by the Ministry of Science and ICT (NRF‐2012R1A1A3008042 and NRF‐2016R1E1A1A01943303).
